# Crystal structure and Hirshfeld surface analysis of (*E*)-4-{2,2-di­chloro-1-[(3,5-di­methyl­phen­yl)diazen­yl]ethen­yl}-*N*,*N*-di­methyl­aniline

**DOI:** 10.1107/S2056989020009202

**Published:** 2020-07-10

**Authors:** Kadriye Özkaraca, Mehmet Akkurt, Namiq Q. Shikhaliyev, Ulviyya F. Askerova, Gulnar T. Suleymanova, Gunay Z. Mammadova, Daniel M. Shadrack

**Affiliations:** a Institute of Natural and Applied Science, Erciyes University, 38039 Kayseri, Turkey; b Department of Physics, Faculty of Sciences, Erciyes University, 38039 Kayseri, Turkey; c Organic Chemistry Department, Baku State University, Z. Khalilov str. 23, AZ, 1148 Baku, Azerbaijan; d Department of Health & Biomedical Sciences, School of Life Science and Bioengineering, The Nelson Mandela Africa Institute of Science and Technology, PO Box 447, Arusha, Tanzania; e Department of Chemistry, St. John’s University of Tanzania, PO Box 47, Dodoma, Tanzania

**Keywords:** crystal structure, Cl⋯Cl halogen bonds, Hirshfeld surface analysis

## Abstract

In the title compound, C_18_H_19_Cl_2_N_3_, the planes of the benzene rings subtend a dihedral angle of 77.07 (10)°. In the crystal, mol­ecules are associated into inversion dimers *via* short Cl⋯Cl contacts [3.3763 (9) Å]. A Hirshfeld surface analysis indicates that the most important contact percentages for the different types of inter­actions are H⋯H (43.9%), Cl⋯H/H⋯Cl (22.9%), C⋯H/H⋯C (20.8%) and N⋯H/H⋯N (8.0%).

## Abstract 2

In the crystal of the title compound, the mol­ecules are associated into inversion dimers *via* short Cl⋯Cl halogen bonds.

## Chemical context   

Aromatic azo compounds provide ubiquitous motifs in organic chemistry and are widely used as organic dyes, indicators, pigments, food additives, ligands, radical reaction initiators, therapeutic agents, *etc*. (Maharramov *et al.*, 2010 ▸; Mahmudov *et al.*, 2013 ▸). On the other hand, the study of both inter- and intra­molecular non-covalent inter­actions in azo compounds is important for our understanding of the factors governing the assembly of the mol­ecules into supra­molecular systems (see, for example, Mahmudov *et al.*, 2015 ▸; Shixaliyev *et al.*, 2014 ▸). When compared to well-explored hydrogen-bonding and *π-*inter­actions (see, for example, Akbari *et al.*, 2017 ▸; Mahmoudi *et al.*, 2018 ▸), the exploration of new inter­molecular inter­actions such as halogen, chalcogen, pnictogen, tetrel and triel bonds is in progress. Thus, decorating the structure of azo compounds with tailored functionalities (halogen, chalcogen and tetrel bond-donor centres) can be an important strategy to control and tune their functional properties such as their analytical and solvatochromic behaviour (Mahmudov *et al.*, 2010 ▸; Mahmudov & Pombeiro, 2016 ▸).
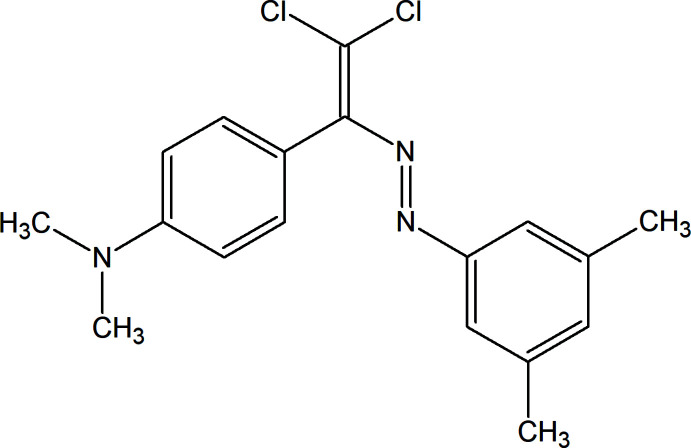


In order to continue our work in this direction, we now describe the synthesis and structure of the title compound, C_18_H_19_Cl_2_N_3_ (I) and its Hirshfeld surface analysis.

## Structural commentary   

**Figure 1:**
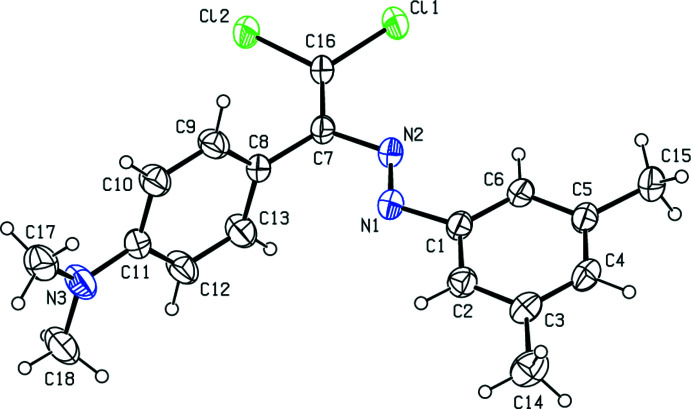
The mol­ecular structure of (I) with displacement ellipsoids drawn at the 30% probability level.

The title compound has a non-planar mol­ecular conformation (Fig. 1 ▸); the dihedral angle between the planes of the C1–C6 and C8–C13 aromatic rings is 77.07 (10)°. The amine N atom as well as the directly adjacent arene C atom are displaced out of the plane of the other five aromatic C atoms: the deviations are −0.009 (2) for C11 and −0.065 (2) Å for N3. Some key torsion angles describing the mol­ecular conformation are C6—C1—N1—N2 [–0.5 (3)°], C1—N1—N2—C7 [–178.40 (15)°], N1—N2—C7—C8 [−6.1 (3)°], N1—N2—C7—C16 [–173.27 (17)°], N2—C7—C8—C13 [–72.1 (3)°], N2—C7—C16—Cl1 [–0.9 (3)°], N2—C7—C16—Cl2 [179.97 (14)°] and C8—C7—C16—Cl2 [–0.6 (3)°]. All of the C=C, N=N, C—Cl bond lengths in (I) are similar to those in the related azocompounds reported in the *Database survey*.

## Supra­molecular features   

**Figure 2:**
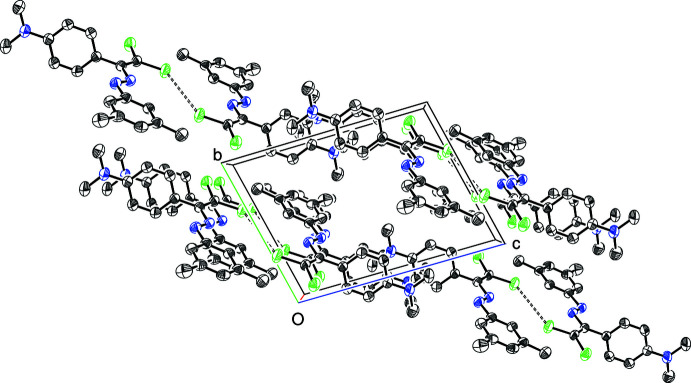
Crystal packing for (I) viewed along the *a*-axis direction.

In the crystal, mol­ecules of (I) are linked into inversion dimers *via* short halogen⋯halogen contacts [Cl1⋯Cl1^i^ = 3.3763 (9) Å C16—Cl1⋯Cl1^i^ = 141.47 (7)°; symmetry code: (i) = 2 – *x*, 1 − *y*, 2 − *z*] compared to the van der Waals radius sum of 3.50 Å. No other directional contacts could be identified and the shortest aromatic-ring-centroid separation is greater than 5.25 Å. The packing for (I) is shown in Fig. 2 ▸.

## Hirshfeld surface analysis   

**Figure 3:**
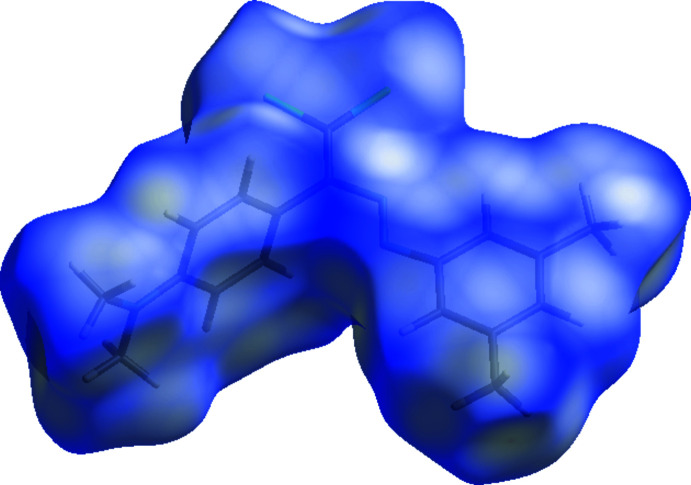
A view of the three-dimensional Hirshfeld surface for (I) plotted over *d*_norm_ in the range −0.07 to 1.33 a.u.

The Hirshfeld surface (McKinnon *et al.*, 2007 ▸) for (I) and its associated two-dimensional fingerprint plots (Spackman & McKinnon, 2002 ▸) were calculated using *CrystalExplorer17* (Turner *et al.*, 2017 ▸). Red, white and blue regions visible on the *d*_norm_ surface indicate contacts with distances shorter, longer and approximately equal to the van der Waals radii: the surface for (I) (Fig. 3 ▸) is almost featureless, indicating a lack of directional inter­actions.

**Figure 4:**
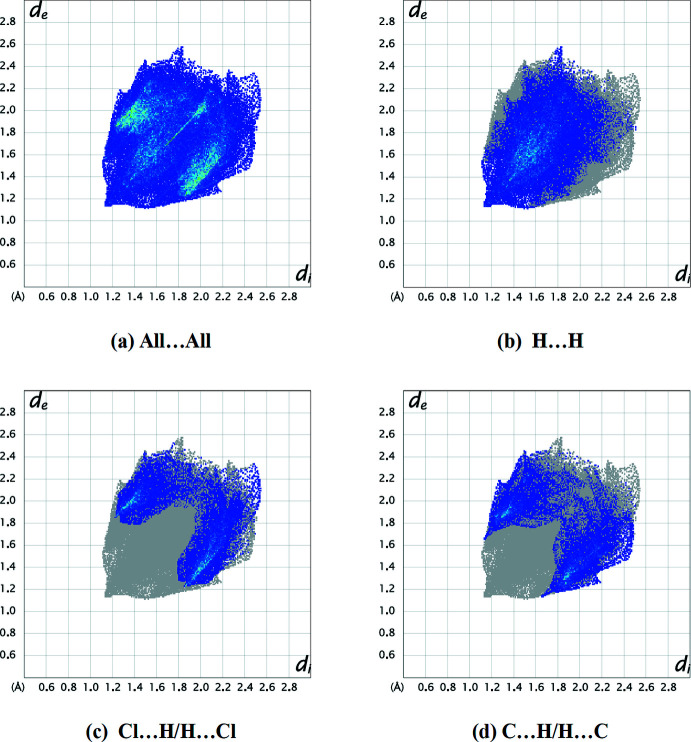
A view of the two-dimensional fingerprint plots for (I) showing (*a*) all inter­actions, and separated into (*b*) H⋯H, (*c*) Cl⋯H/H⋯Cl and (*d*) C⋯H/H⋯C inter­actions. The *d*_i_ and *d*_e_ values are the closest inter­nal and external distances (in Å) from given points on the Hirshfeld surface contacts.

The overall two-dimensional fingerprint plot (Fig. 4 ▸ *a*) and those delineated into H⋯H, Cl⋯H/H⋯Cl and C⋯H/H⋯C contacts (McKinnon *et al.*, 2007 ▸) are illustrated in Fig. 4 ▸ *b*–*d*, respectively and percentage contributions to the Hirshfeld surface are given in Table 1 ▸. The most important inter­action is H⋯H, contributing 43.9% to the overall surface, which is reflected in Fig. 4 ▸ *b* as widely scattered points of high density due to the large hydrogen content of the mol­ecule, with the tip at *d*_e_ = *d*_i_ = 1.15 Å. The reciprocal Cl⋯H/H⋯Cl inter­actions appear as two symmetrical broad wings with *d*_e_ + *d*_i_ ≃ 3.05 Å and contribute 22.9% to the Hirshfeld surface (Fig. 4 ▸ *c*). The pair of characteristic wings in the fingerprint plot delineated into C⋯H/H⋯C contacts (Fig. 4 ▸ *d*; 20.8% contribution to the Hirshfeld surface), have the tips at *d*_e_ + *d*_i_ ≃ 2.80 Å. The remaining contributions from the other different inter­atomic contacts to the Hirshfeld surfaces are listed in Table 1 ▸. The small contribution of the other weak inter­molecular N⋯H/H⋯N, Cl⋯C/C⋯Cl, Cl⋯Cl, N⋯C/C⋯N and C⋯C contacts suggest a negligible effect on the packing. The dominance of H-atom contacts suggest that van der Waals inter­actions play the major role in establishing the crystal packing for (I) (Hathwar *et al.*, 2015 ▸).

## Database survey   

A search of the Cambridge Structural Database (CSD, Version 5.41, update of November 2019; Groom *et al.*, 2016 ▸) the (*E*)-1-(2,2-di­chloro-1-phenyl­ethen­yl)-2-phenyl­diazene unit resulted in 25 hits. Six compounds are closely related to the title compound, *viz*. 1-(4-bromo­phen­yl)-2-[2,2-di­chloro-1-(4-nitro­phen­yl)ethen­yl]diazene (CSD refcode HONBOE; Akkurt *et al.*, 2019 ▸), 1-(4-chloro­phen­yl)-2-[2,2-di­chloro-1-(4-nitro­phen­yl)ethen­yl]diazene (HONBUK; Akkurt *et al.*, 2019 ▸), 1-(4-chloro­phen­yl)-2-[2,2-di­chloro-1-(4-fluoro­phen­yl)ethen­yl]diazene (HODQAV; Shikhaliyev *et al.*, 2019 ▸), 1-[2,2-di­chloro-1-(4-nitro­phen­yl)ethen­yl]-2-(4-fluoro­phen­yl)diazene (XIZREG; Atioğlu *et al.*, 2019 ▸), 1,1-[methyl­enebis(4,1-phenyl­ene)]bis­[(2,2-di­chloro-1-(4-nitro­phen­yl)ethen­yl]diaz­ene (LEQXIR; Shikhaliyev *et al.*, 2018 ▸) and 1,1-[methyl­enebis(4,1-phenyl­ene)]bis­{[2,2-di­chloro-1-(4-chloro­phen­yl) ethen­yl]diazene} (LEQXOX; Shikhaliyev *et al.*, 2018 ▸).

In the crystals of HONBOE and HONBUK, the aromatic rings form dihedral angles of 60.9 (2) and 64.1 (2)°, respectively. Mol­ecules are linked through weak *X*⋯Cl contacts (*X* = Br for HONBOE and Cl for HONBUK), C—H⋯Cl and C—Cl⋯π inter­actions into sheets parallel to the *ab* plane. Additional van der Waals inter­actions consolidate the three-dimensional packing. In the crystal of HODQAV, the planes of the benzene rings make a dihedral angle of 56.13 (13)°. Mol­ecules are stacked in columns along the *a*-axis direction *via* weak C—H⋯Cl hydrogen bonds and face-to-face π–π stacking inter­actions. The crystal packing is further consolidated by short Cl⋯Cl contacts. In XIZREG, the benzene rings form a dihedral angle of 63.29 (8)°. Mol­ecules are linked by C—H⋯O hydrogen bonds into zigzag chains running along the *c*-axis direction. The crystal packing also features C—Cl⋯π, C—F⋯π and N—O⋯π inter­actions. In the crystals of LEQXIR and LEQXOX, the dihedral angles between the aromatic rings are 56.18 (12) and 60.31 (14)°, respectively. In LEQXIR, C—H⋯N and C—H⋯O hydrogen bonds and short Cl⋯O contacts occur and in LEQXOX C—H⋯N and short Cl⋯Cl contacts are observed.

## Synthesis and crystallization   

A 20 ml screw-neck vial was charged with DMSO (10 ml), (*Z*)-4-{[2-(3,5-di­methyl­phen­yl)hydrazineyl­idene]meth­yl}-*N,N*-di­methyl­aniline (267 mg, 1.00 mmol), tetra­methyl­ethylenedi­amine (TMEDA) (295 mg, 2.50 mmol), CuCl (2 mg, 0.02 mmol) and CCl_4_ (20 mmol, 10 equiv). After 1–3 h (until TLC analysis showed complete consumption of the corres­ponding Schiff base) the reaction mixture was poured into ∼0.01 *M* solution of HCl (100 ml, pH = 2–3) and extracted with di­chloro­methane (3 × 20 ml). The combined organic phase was washed with water (3 × 50 ml), brine (30 ml), dried over anhydrous Na_2_SO_4_ and concentrated *in vacuo* using a rotary evaporator. The residue was purified by column chromatography on silica gel using appropriate mixtures of hexane and di­chloro­methane (3:1–1:1) to form a red solid in 85% yield (m.p. 429 K). Orange plates of (I) were obtained by the slow evaporation of an ethanol solution. Analysis calculated for C_18_H_19_Cl_2_N_3_: C 62.08, H 5.50, N 12.07; found: C 62.01, H 5.48, N 12.03%. ^1^H NMR (300 MHz, CDCl_3_) *δ* 2.38 (6H, ArMe_2_), 3.05 (6H, NMe_2_), 6.88–7.43 (7H, Ar). ^13^C NMR (75MHz, CDCl_3_) δ 155.57, 153.15, 151.94, 147.03, 142.69, 138.64, 137.97, 133.14, 131.20, 127.08, 121.02, 21.20. ESI–MS: *m*/*z*: 349.18 [*M*+H]^+^.

## Refinement   

Crystal data, data collection and structure refinement details are summarized in Table 2 ▸. All C-bound H atoms were placed in idealized locations and refined using a riding model with C—H = 0.93–0.96 Å. The constraint *U*_iso_(H) = 1.2U_eq_(C) or 1.5*U*_eq_(methyl C) was applied in all cases.

## Supplementary Material

Crystal structure: contains datablock(s) I. DOI: 10.1107/S2056989020009202/hb7912sup1.cif

Structure factors: contains datablock(s) I. DOI: 10.1107/S2056989020009202/hb7912Isup2.hkl

CCDC reference: 2014419

Additional supporting information:  crystallographic information; 3D view; checkCIF report

## Figures and Tables

**Table 1:** Percentage contributions of inter­atomic contacts to the Hirshfeld surface for (I)

Contact	Percentage contribution
H⋯H	43.9
Cl⋯H/H⋯Cl	22.9
C⋯H/H⋯C	20.8
N⋯H/H⋯N	8.0
Cl⋯C/C⋯Cl	2.3
Cl⋯Cl	1.4
N⋯C/C⋯N	0.3
C⋯C	0.3

**Table 2:** Experimental details

Crystal data	
Chemical formula	C_18_H_19_Cl_2_N_3_
*M*_r_	348.26
Crystal system, space group	Triclinic, *P* 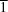
Temperature (K)	296
*a*, *b*, *c* (Å)	8.1035 (4), 9.1965 (5), 12.3665 (7)
α, β, γ (°)	102.421 (2), 95.880 (2), 91.105 (2)
*V* (Å^3^)	894.48 (8)
*Z*	2
Radiation type	Mo *K*α
μ (mm^−1^)	0.37
Crystal size (mm)	0.28 × 0.22 × 0.18

Data collection	
Diffractometer	Bruker APEXII CCD
Absorption correction	Multi-scan (*SADABS*; Krause *et al.*, 2015 ▸)
*T*_min_, *T*_max_	0.897, 0.924
No. of measured, independent and observed [*I* > 2σ(*I*)] reflections	13675, 3339, 2786
*R*_int_	0.039
(sin θ/λ)_max_ (Å^−1^)	0.611

Refinement	
*R*[*F* ^2^ > 2σ(*F* ^2^)], *wR*(*F* ^2^), *S*	0.045, 0.125, 1.03
No. of reflections	3339
No. of parameters	212
H-atom treatment	H-atom parameters constrained
Δρ_max_, Δρ_min_ (e Å^−3^)	0.30, −0.21

Computer programs: *APEX3* and *SAINT* (Bruker, 2007 ▸), *SHELXS* (Sheldrick, 2008 ▸), *SHELXL2016/6* (Sheldrick, 2015 ▸), *ORTEP-3 for Windows* (Farrugia, 2012 ▸) and *PLATON* (Spek, 2020 ▸).

## supplementary crystallographic information

### Crystal data

**Table:**

C_18_H_19_Cl_2_N_3_	*Z* = 2
*M**_r_* = 348.26	*F*(000) = 364
Triclinic, *P*1	*D*_x_ = 1.293 Mg m^−^^3^
*a* = 8.1035 (4) Å	Mo *K*α radiation, λ = 0.71073 Å
*b* = 9.1965 (5) Å	Cell parameters from 7784 reflections
*c* = 12.3665 (7) Å	θ = 2.3–25.7°
α = 102.421 (2)°	µ = 0.37 mm^−^^1^
β = 95.880 (2)°	*T* = 296 K
γ = 91.105 (2)°	Plate, orange
*V* = 894.48 (8) Å^3^	0.28 × 0.22 × 0.18 mm

### Data collection

**Table:**

Bruker APEXII CCD diffractometer	2786 reflections with *I* > 2σ(*I*)
φ and ω scans	*R*_int_ = 0.039
Absorption correction: multi-scan (SADABS; Krause *et al.*, 2015)	θ_max_ = 25.8°, θ_min_ = 2.3°
*T*_min_ = 0.897, *T*_max_ = 0.924	*h* = −9→9
13675 measured reflections	*k* = −10→11
3339 independent reflections	*l* = −15→15

### Refinement

**Table:**

Refinement on *F*^2^	Primary atom site location: structure-invariant direct methods
Least-squares matrix: full	Hydrogen site location: inferred from neighbouring sites
*R*[*F*^2^ > 2σ(*F*^2^)] = 0.045	H-atom parameters constrained
*wR*(*F*^2^) = 0.125	*w* = 1/[σ^2^(*F*_o_^2^) + (0.0539*P*)^2^ + 0.3599*P*] where *P* = (*F*_o_^2^ + 2*F*_c_^2^)/3
*S* = 1.03	(Δ/σ)_max_ = 0.001
3339 reflections	Δρ_max_ = 0.30 e Å^−^^3^
212 parameters	Δρ_min_ = −0.21 e Å^−^^3^
0 restraints	

### Special details

**Table:**

Geometry. All esds (except the esd in the dihedral angle between two l.s. planes) are estimated using the full covariance matrix. The cell esds are taken into account individually in the estimation of esds in distances, angles and torsion angles; correlations between esds in cell parameters are only used when they are defined by crystal symmetry. An approximate (isotropic) treatment of cell esds is used for estimating esds involving l.s. planes.

### Fractional atomic coordinates and isotropic or equivalent isotropic displacement parameters (Å^2^)

**Table:**

	*x*	*y*	*z*	*U*_iso_*/*U*_eq_	
C1	0.4932 (2)	0.5319 (2)	0.78212 (16)	0.0507 (4)	
C2	0.3377 (3)	0.5251 (2)	0.72287 (17)	0.0572 (5)	
H2A	0.313279	0.592879	0.678105	0.069*	
C3	0.2180 (2)	0.4186 (2)	0.72940 (19)	0.0586 (5)	
C4	0.2585 (2)	0.3198 (2)	0.79718 (18)	0.0578 (5)	
H4A	0.179261	0.247628	0.801995	0.069*	
C5	0.4136 (2)	0.3245 (2)	0.85843 (16)	0.0528 (4)	
C6	0.5313 (2)	0.4323 (2)	0.85097 (16)	0.0515 (4)	
H6A	0.635267	0.438287	0.891630	0.062*	
C7	0.8563 (2)	0.7644 (2)	0.81327 (15)	0.0486 (4)	
C8	0.8188 (2)	0.8616 (2)	0.73299 (15)	0.0470 (4)	
C9	0.7799 (3)	1.0079 (2)	0.76813 (17)	0.0624 (5)	
H9A	0.780539	1.047283	0.844019	0.075*	
C10	0.7401 (3)	1.0978 (2)	0.69421 (18)	0.0663 (6)	
H10A	0.713687	1.195886	0.721428	0.080*	
C11	0.7385 (2)	1.0455 (2)	0.58013 (16)	0.0530 (4)	
C12	0.7818 (4)	0.8986 (3)	0.54516 (18)	0.0731 (7)	
H12A	0.785747	0.859575	0.469609	0.088*	
C13	0.8188 (3)	0.8099 (2)	0.61981 (18)	0.0705 (6)	
H13A	0.844835	0.711493	0.593078	0.085*	
C14	0.0476 (3)	0.4121 (3)	0.6668 (3)	0.0815 (7)	
H14A	0.015539	0.310513	0.631791	0.122*	
H14B	0.049299	0.470111	0.610925	0.122*	
H14C	−0.030567	0.451581	0.717658	0.122*	
C15	0.4523 (3)	0.2146 (3)	0.9306 (2)	0.0681 (6)	
H15A	0.553126	0.246587	0.978497	0.102*	
H15B	0.465759	0.118284	0.884317	0.102*	
H15C	0.362933	0.208661	0.974908	0.102*	
C16	0.9971 (2)	0.7786 (2)	0.88186 (16)	0.0520 (4)	
C17	0.6238 (4)	1.2751 (3)	0.5403 (2)	0.0806 (7)	
H17A	0.556425	1.271764	0.599308	0.121*	
H17B	0.556809	1.299516	0.478616	0.121*	
H17C	0.711966	1.349476	0.566573	0.121*	
C18	0.6996 (5)	1.0776 (3)	0.3882 (2)	0.0944 (9)	
H18A	0.639013	0.983527	0.364268	0.142*	
H18B	0.813161	1.065188	0.373532	0.142*	
H18C	0.651020	1.147779	0.348239	0.142*	
Cl1	1.04137 (8)	0.67088 (7)	0.97728 (5)	0.0734 (2)	
Cl2	1.15092 (7)	0.90973 (7)	0.88372 (5)	0.0707 (2)	
N1	0.6056 (2)	0.64606 (18)	0.76759 (14)	0.0552 (4)	
N2	0.7438 (2)	0.64937 (17)	0.82395 (13)	0.0523 (4)	
N3	0.6932 (3)	1.1321 (2)	0.50518 (15)	0.0745 (6)	

### Atomic displacement parameters (Å^2^)

**Table:**

	*U*^11^	*U*^22^	*U*^33^	*U*^12^	*U*^13^	*U*^23^
C1	0.0541 (10)	0.0443 (9)	0.0535 (10)	−0.0068 (8)	0.0080 (8)	0.0098 (8)
C2	0.0576 (11)	0.0520 (11)	0.0617 (12)	0.0014 (8)	0.0037 (9)	0.0127 (9)
C3	0.0487 (10)	0.0522 (11)	0.0701 (12)	−0.0004 (8)	0.0069 (9)	0.0031 (9)
C4	0.0515 (11)	0.0497 (11)	0.0695 (13)	−0.0095 (8)	0.0145 (9)	0.0044 (9)
C5	0.0587 (11)	0.0445 (10)	0.0555 (11)	−0.0058 (8)	0.0135 (9)	0.0085 (8)
C6	0.0522 (10)	0.0489 (10)	0.0524 (10)	−0.0075 (8)	0.0047 (8)	0.0104 (8)
C7	0.0573 (10)	0.0433 (9)	0.0470 (9)	−0.0069 (8)	0.0098 (8)	0.0127 (7)
C8	0.0526 (10)	0.0438 (9)	0.0462 (9)	−0.0061 (7)	0.0053 (7)	0.0139 (7)
C9	0.0852 (15)	0.0571 (12)	0.0450 (10)	0.0091 (10)	0.0075 (10)	0.0105 (9)
C10	0.0933 (16)	0.0507 (11)	0.0555 (11)	0.0152 (11)	0.0078 (11)	0.0116 (9)
C11	0.0605 (11)	0.0506 (10)	0.0509 (10)	0.0002 (8)	0.0071 (8)	0.0173 (8)
C12	0.116 (2)	0.0619 (13)	0.0454 (11)	0.0188 (13)	0.0177 (11)	0.0156 (9)
C13	0.1110 (19)	0.0516 (11)	0.0516 (11)	0.0172 (12)	0.0162 (11)	0.0124 (9)
C14	0.0548 (13)	0.0765 (16)	0.107 (2)	0.0020 (11)	−0.0048 (12)	0.0125 (14)
C15	0.0748 (14)	0.0611 (13)	0.0729 (14)	−0.0112 (10)	0.0133 (11)	0.0237 (11)
C16	0.0617 (11)	0.0470 (10)	0.0501 (10)	−0.0114 (8)	0.0049 (8)	0.0187 (8)
C17	0.0918 (18)	0.0726 (15)	0.0815 (16)	0.0185 (13)	−0.0053 (13)	0.0312 (13)
C18	0.147 (3)	0.0867 (18)	0.0594 (14)	0.0169 (18)	0.0137 (15)	0.0345 (13)
Cl1	0.0839 (4)	0.0724 (4)	0.0705 (4)	−0.0174 (3)	−0.0110 (3)	0.0408 (3)
Cl2	0.0679 (4)	0.0737 (4)	0.0743 (4)	−0.0273 (3)	−0.0064 (3)	0.0340 (3)
N1	0.0584 (10)	0.0498 (9)	0.0589 (9)	−0.0091 (7)	0.0039 (8)	0.0170 (7)
N2	0.0568 (9)	0.0478 (8)	0.0543 (9)	−0.0093 (7)	0.0075 (7)	0.0161 (7)
N3	0.1083 (16)	0.0659 (11)	0.0564 (10)	0.0201 (11)	0.0110 (10)	0.0269 (9)

### Geometric parameters (Å, º)

**Table:**

C1—C2	1.384 (3)	C11—C12	1.391 (3)
C1—C6	1.397 (3)	C12—C13	1.373 (3)
C1—N1	1.429 (2)	C12—H12A	0.9300
C2—C3	1.385 (3)	C13—H13A	0.9300
C2—H2A	0.9300	C14—H14A	0.9600
C3—C4	1.385 (3)	C14—H14B	0.9600
C3—C14	1.506 (3)	C14—H14C	0.9600
C4—C5	1.394 (3)	C15—H15A	0.9600
C4—H4A	0.9300	C15—H15B	0.9600
C5—C6	1.387 (3)	C15—H15C	0.9600
C5—C15	1.503 (3)	C16—Cl1	1.7123 (19)
C6—H6A	0.9300	C16—Cl2	1.7129 (18)
C7—C16	1.336 (3)	C17—N3	1.438 (3)
C7—N2	1.420 (2)	C17—H17A	0.9600
C7—C8	1.485 (2)	C17—H17B	0.9600
C8—C9	1.375 (3)	C17—H17C	0.9600
C8—C13	1.378 (3)	C18—N3	1.433 (3)
C9—C10	1.378 (3)	C18—H18A	0.9600
C9—H9A	0.9300	C18—H18B	0.9600
C10—C11	1.388 (3)	C18—H18C	0.9600
C10—H10A	0.9300	N1—N2	1.254 (2)
C11—N3	1.374 (3)		
			
C2—C1—C6	120.38 (17)	C12—C13—C8	122.3 (2)
C2—C1—N1	115.34 (17)	C12—C13—H13A	118.9
C6—C1—N1	124.28 (17)	C8—C13—H13A	118.9
C1—C2—C3	120.85 (19)	C3—C14—H14A	109.5
C1—C2—H2A	119.6	C3—C14—H14B	109.5
C3—C2—H2A	119.6	H14A—C14—H14B	109.5
C4—C3—C2	118.00 (19)	C3—C14—H14C	109.5
C4—C3—C14	120.8 (2)	H14A—C14—H14C	109.5
C2—C3—C14	121.2 (2)	H14B—C14—H14C	109.5
C3—C4—C5	122.48 (18)	C5—C15—H15A	109.5
C3—C4—H4A	118.8	C5—C15—H15B	109.5
C5—C4—H4A	118.8	H15A—C15—H15B	109.5
C6—C5—C4	118.55 (18)	C5—C15—H15C	109.5
C6—C5—C15	120.77 (19)	H15A—C15—H15C	109.5
C4—C5—C15	120.67 (18)	H15B—C15—H15C	109.5
C5—C6—C1	119.72 (18)	C7—C16—Cl1	124.05 (14)
C5—C6—H6A	120.1	C7—C16—Cl2	122.70 (14)
C1—C6—H6A	120.1	Cl1—C16—Cl2	113.24 (11)
C16—C7—N2	114.49 (16)	N3—C17—H17A	109.5
C16—C7—C8	123.12 (16)	N3—C17—H17B	109.5
N2—C7—C8	122.39 (16)	H17A—C17—H17B	109.5
C9—C8—C13	116.53 (18)	N3—C17—H17C	109.5
C9—C8—C7	121.42 (17)	H17A—C17—H17C	109.5
C13—C8—C7	122.04 (17)	H17B—C17—H17C	109.5
C8—C9—C10	121.94 (18)	N3—C18—H18A	109.5
C8—C9—H9A	119.0	N3—C18—H18B	109.5
C10—C9—H9A	119.0	H18A—C18—H18B	109.5
C9—C10—C11	121.60 (19)	N3—C18—H18C	109.5
C9—C10—H10A	119.2	H18A—C18—H18C	109.5
C11—C10—H10A	119.2	H18B—C18—H18C	109.5
N3—C11—C10	122.27 (19)	N2—N1—C1	113.12 (16)
N3—C11—C12	121.46 (18)	N1—N2—C7	114.22 (16)
C10—C11—C12	116.26 (18)	C11—N3—C18	121.15 (19)
C13—C12—C11	121.38 (19)	C11—N3—C17	121.07 (19)
C13—C12—H12A	119.3	C18—N3—C17	117.6 (2)
C11—C12—H12A	119.3		
			
C6—C1—C2—C3	−0.9 (3)	C9—C10—C11—C12	−1.0 (4)
N1—C1—C2—C3	179.58 (18)	N3—C11—C12—C13	−176.6 (2)
C1—C2—C3—C4	0.2 (3)	C10—C11—C12—C13	1.9 (4)
C1—C2—C3—C14	178.9 (2)	C11—C12—C13—C8	−1.4 (4)
C2—C3—C4—C5	0.2 (3)	C9—C8—C13—C12	−0.2 (4)
C14—C3—C4—C5	−178.5 (2)	C7—C8—C13—C12	179.0 (2)
C3—C4—C5—C6	0.0 (3)	N2—C7—C16—Cl1	−0.9 (3)
C3—C4—C5—C15	−179.74 (19)	C8—C7—C16—Cl1	178.48 (14)
C4—C5—C6—C1	−0.6 (3)	N2—C7—C16—Cl2	179.97 (14)
C15—C5—C6—C1	179.08 (18)	C8—C7—C16—Cl2	−0.6 (3)
C2—C1—C6—C5	1.1 (3)	C2—C1—N1—N2	179.06 (17)
N1—C1—C6—C5	−179.40 (17)	C6—C1—N1—N2	−0.5 (3)
C16—C7—C8—C9	−72.4 (3)	C1—N1—N2—C7	−178.40 (15)
N2—C7—C8—C9	107.0 (2)	C16—C7—N2—N1	−173.27 (17)
C16—C7—C8—C13	108.5 (3)	C8—C7—N2—N1	−6.1 (3)
N2—C7—C8—C13	−72.1 (3)	C10—C11—N3—C18	177.2 (3)
C13—C8—C9—C10	1.1 (3)	C12—C11—N3—C18	−4.4 (4)
C7—C8—C9—C10	−178.0 (2)	C10—C11—N3—C17	−8.3 (4)
C8—C9—C10—C11	−0.5 (4)	C12—C11—N3—C17	170.1 (3)
C9—C10—C11—N3	177.5 (2)		
